# Randomized controlled trials of vitamin D and cancer incidence: A modeling study

**DOI:** 10.1371/journal.pone.0176448

**Published:** 2017-05-01

**Authors:** William B. Grant, Barbara J. Boucher

**Affiliations:** 1 Sunlight, Nutrition, and Health Research Center, San Francisco, California, United States of America; 2 Blizard Institute, Barts & The London School of Medicine & Dentistry, Queen Mary University of London, London, United Kingdom; University of Alabama at Birmingham, UNITED STATES

## Abstract

Although geographic ecological studies and observational studies find that ultraviolet B exposure and 25-hydroxyvitamin D [25(OH)D] concentrations are inversely correlated with 15–20 types of cancer, few randomized controlled trials (RCTs) of vitamin D support those findings. The poor design of some RCTs may account for that lack of support. Most vitamin D RCTs to date have considered the vitamin D dose, rather than initial, final, or changes in, serum 25(OH)D concentrations. Here a model is developed for use in designing and analyzing vitamin D RCTs with application to cancer incidence. The input variables of the model are vitamin D dose, baseline and achieved 25(OH)D concentrations, known rates of cancer for the population, and numbers of participants for the treatment and placebo arms is estimated—vitamin D dosage and numbers of participants are varied to achieve desired hazard ratio significance, using information from two vitamin D RCTs on cancer incidence conducted in Nebraska with good agreement between the model estimates and reported hazard ratios. Further improvements to the conduct of vitamin D RCTs would be to start the trial with a moderate bolus dose to achieve the desired 25(OH)D concentrations, and bloodspot 25(OH)D assay use in summer and winter annually to monitor seasonal and long-term changes in 25(OH)D concentration and compliance, and to allow dosage adjustment for achievement of desired vitamin D status.

## Introduction

Many important health benefits are known to be associated with higher 25-hydroxyvitamin D [25(OH)D] concentrations, including reduced risk of many cancers [1],[2]. The Garland brothers first proposed a role for vitamin D in reducing cancer risk on the basis of a geographical ecological study of colon cancer mortality rates and annual solar radiation doses [3]. From the findings of single-country ecological studies, 15–20 cancers have incidence and/or mortality rates inversely correlated with solar UVB doses [1]. From observational studies, there are clear inverse correlations between serum 25(OH)D concentration and incidence of several cancers, including breast [4], colorectal [4], kidney [5], lung [6], pancreatic [7], upper aerodigestive [8], and total, cancer incidence [9]. The mechanisms through which vitamin D reduces risk of cancer incidence and increase survival are well known [1],[10].

However, confirming causality of the link between vitamin D and cancer riskrequires randomized controlled trials (RCTs) of vitamin D supplementation. Unfortunately, vitamin D RCTs, in general, do not support observational studies [11],[12]. To date, vitamin D supplementation reduced cancer risk in only three RCTs: two studies involving postmenopausal women in Nebraska [13],[14], and the Women’s Health Initiative, when the analysis was restricted to participants who had not taken vitamin D or calcium supplements before entering the trial [15].

Robert Heaney pointed out that the improper design of vitamin D RCTs accounts leads to their predictable failure to confirm the results of observational studies, since their design was based on standard RCT models for pharmaceuticals rather than on guidelines appropriate for nutrients [16], e.g., the drug model assumes that the only source of the agent is in the trial and that a linear dose–response relationship exists. However, vitamin D trials can satisfy neither assumption. Also, it is not vitamin D dose, per se, that affects health outcomes but rather vitamin D status over time [serum concentrations of 25(OH)D] and, to a lesser extent, serum 1,25-dihydroxyvitamin D. Heaney’s guidelines, as applied to vitamin D, include:

- Ensure an understanding of the 25(OH)D concentration–health outcome relation of interest.
- Measure 25(OH)D concentrations of prospective participants.
- Aim to include only those with 25(OH)D concentrations near the low end of the relationship.
- Supplement those in the treatment arm with enough vitamin D_3_ to increase 25(OH)D concentrations to the upper region of the relationship.
- Measure achieved 25(OH)D concentrations.
- Optimize conutrient status (such as calcium).

We here expand upon Heaney’s guidelines for vitamin D RCTs for cancer, starting with a modeling study, followed by a comparison with results of two cancer vitamin D trials to date, and end by identifying appropriate ways to conduct such trials. The model developed uses existing data on baseline and achieved 25(OH)D concentrations, expected cancer incidence rates as a function of baseline and achieved 25(OH)D concentrations, and calculations of 95% confidence intervals (CIs) for various equal numbers of participant years in treatment and control arms.

## Materials and methods

A model is developed based on the use of population distributions of baseline 25(OH)D concentrations, vitamin D_3_ dosage used, and achieved 25(OH)D concentrations post-supplementation; looking at expected cancer cases for the population as a function of 25(OH)D concentration, and the odds ratios for the effects of vitamin D_3_ supplementation as a function of the number of participant-years in treatment and control arms. This model is then evaluated by comparison of its predictions with findings from two RCTs showing benefits of vitamin D_3_ supplementation on cancer incidence.

**Fig 1:**
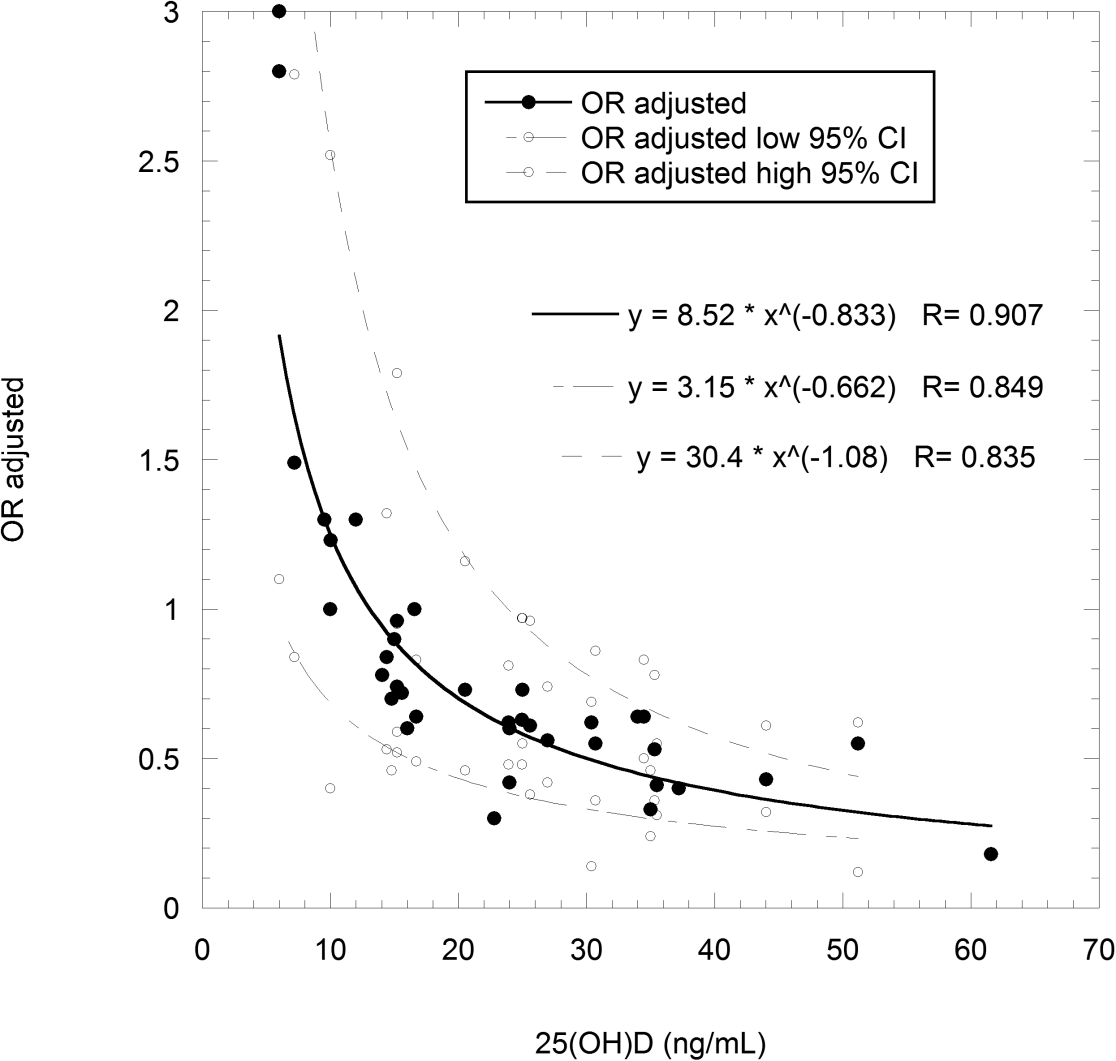
Graph of odds ratio for breast cancer incidence vs. 25(OH)D concentration from 11 case-control studies.

The 25(OH)D concentration–cancer incidence relation used is based on 11 breast cancer case–control studies from seven countries [4], and the relationship is shown in Fig 1. The present model for that data finds an odds ratio (OR) = 18.3×[25(OH)D]^–0.833^. This value differs slightly from that in Fig 2 in [4] since in that figure, one nested case-control study was inadvertently included.

Many authors consider that case–control study findings may be confounded by reverse causation—i.e., low 25(OH)D concentration’s being a result of the life-style, or of physiological disturbances associated with the disease, rather than a cause of the disease [11]. However, the results from 11 studies agree well, and odds ratios for case–control studies for breast and colorectal cancer align with a linear fit to the results from prospective studies with results plotted as a function of follow-up time. Both those facts argue against that contention [4]. That the results for breast cancer can be used as a proxy for all-cancer incidence is supported by the finding that in ecological studies of cancer mortality rates in the U.S. for two periods, 1950–1969 and 1970–1994, the correlations with UVB doses are similar [17]. Also, reductions in incidence of total, breast, colorectal, and invasive breast cancers in the Women's Health Initiative study for women not taking vitamin D or calcium supplements prior to entry were similar [15]. However, should such differences emerge, this modeling study allows utilization of the available data.

The population distributions of serum 25(OH)D concentrations were obtained from a cross-sectional study of Canadians [18]. The values for those aged 50–79 years are used in this paper as given in Table 3 of [19]. Although Canada is north of the U.S., the two countries have similar population means for serum 25(OH)D concentrations: ~24–25 ng/mL (for SI units, in nmol/L, multiply by 2.5) for the U.S. [20], compared with ~24 ng/mL for Canada.

The model developed uses data on baseline and achieved 25(OH)D concentrations, expected cancer incidence rates as a function of 25(OH)D concentration, and calculations of the 95% confidence intervals (CIs) for various equal numbers of participant years in the treatment and control arms.

Achieved 25(OH)D concentrations were estimated using the data developed by GrassrootsHealth from 25(OH)D measurements on 3667 community-based participants in their voluntary measurement program [21], where participants who had not been supplementing with vitamin D prior to entry had a 25(OH)D concentration measurement made [by blood-spot testing], before starting supplementation with vitamin D_3_ and had 25(OH)D concentration re-measured after six months. The best fit curve for their data is shown in their Fig 3 [21]. The rise in 25(OH)D per 1000 IU/d of daily oral vitamin D_3_ decreases from 13 ng/mL for a baseline 25(OH)D of zero, to 4 ng/mL for a baseline 25(OH)D of 28 ng/mL, and to 2 ng/mL at 140 ng/mL. The reason that serum 25(OH)D concentration increases less on any specific dose, with higher basal 25(OH)D concentrations, is that expression and activity, of CYP24A1, the homeostatic enzyme that destroys 25(OH)D and calcitriol, increases with increases in serum 25(OH)D [22].

**Fig 2:**
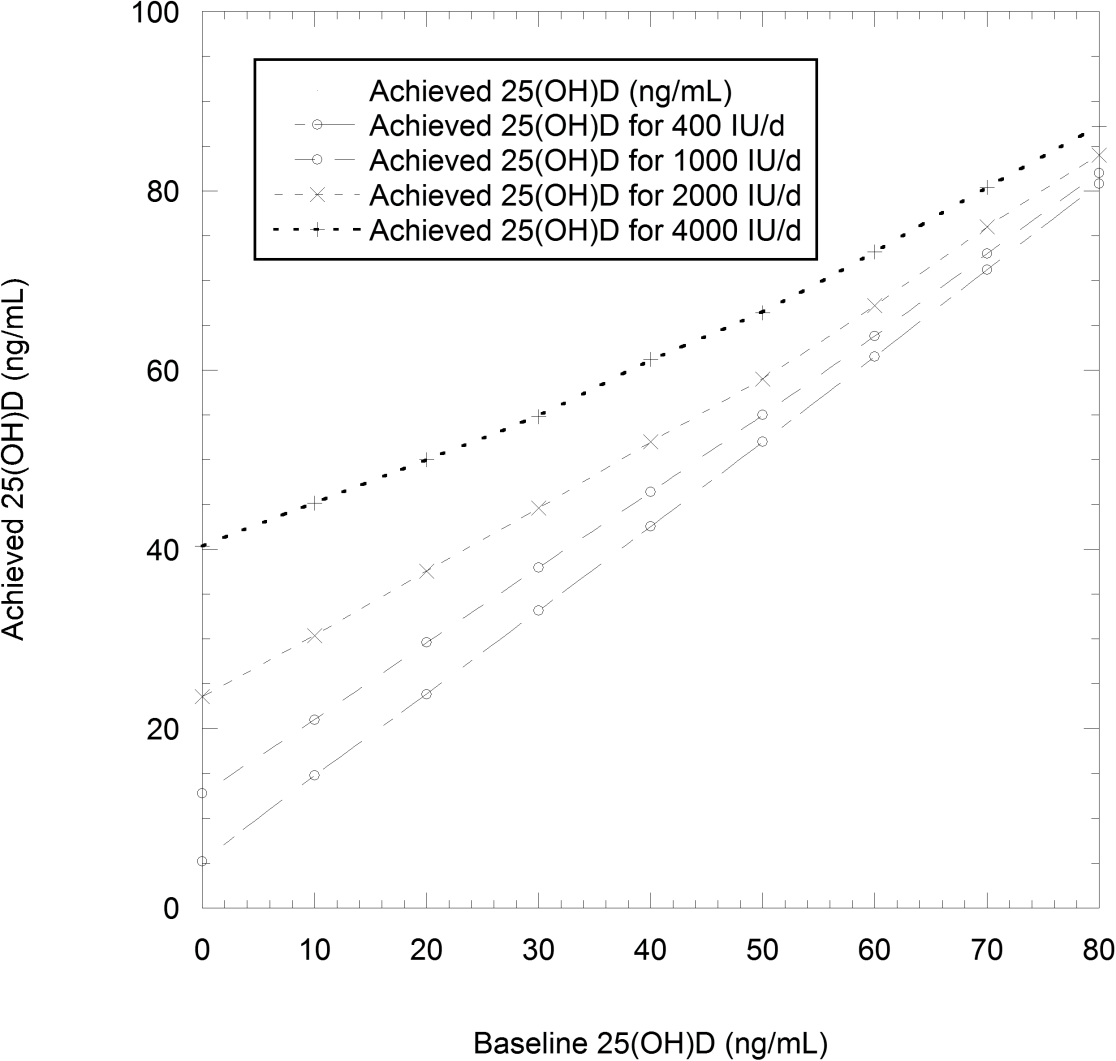
Achieved 25-hydroxvitamin D [25(OH)D] concentration as a function of baseline 25(OH)D concentration, and vitamin D_3_ dosages, as shown in Table 4.

Table 1 presents the estimates of achieved 25(OH)D concentrations for each of the ten deciles of baseline 25(OH)D concentration found for Canadians aged 50–79 years, as predicted for supplementation with 400, 1000, 2000, or 4000 IU/d of vitamin D_3,_ and Fig 2 shows those results graphically.

**Fig 3:**
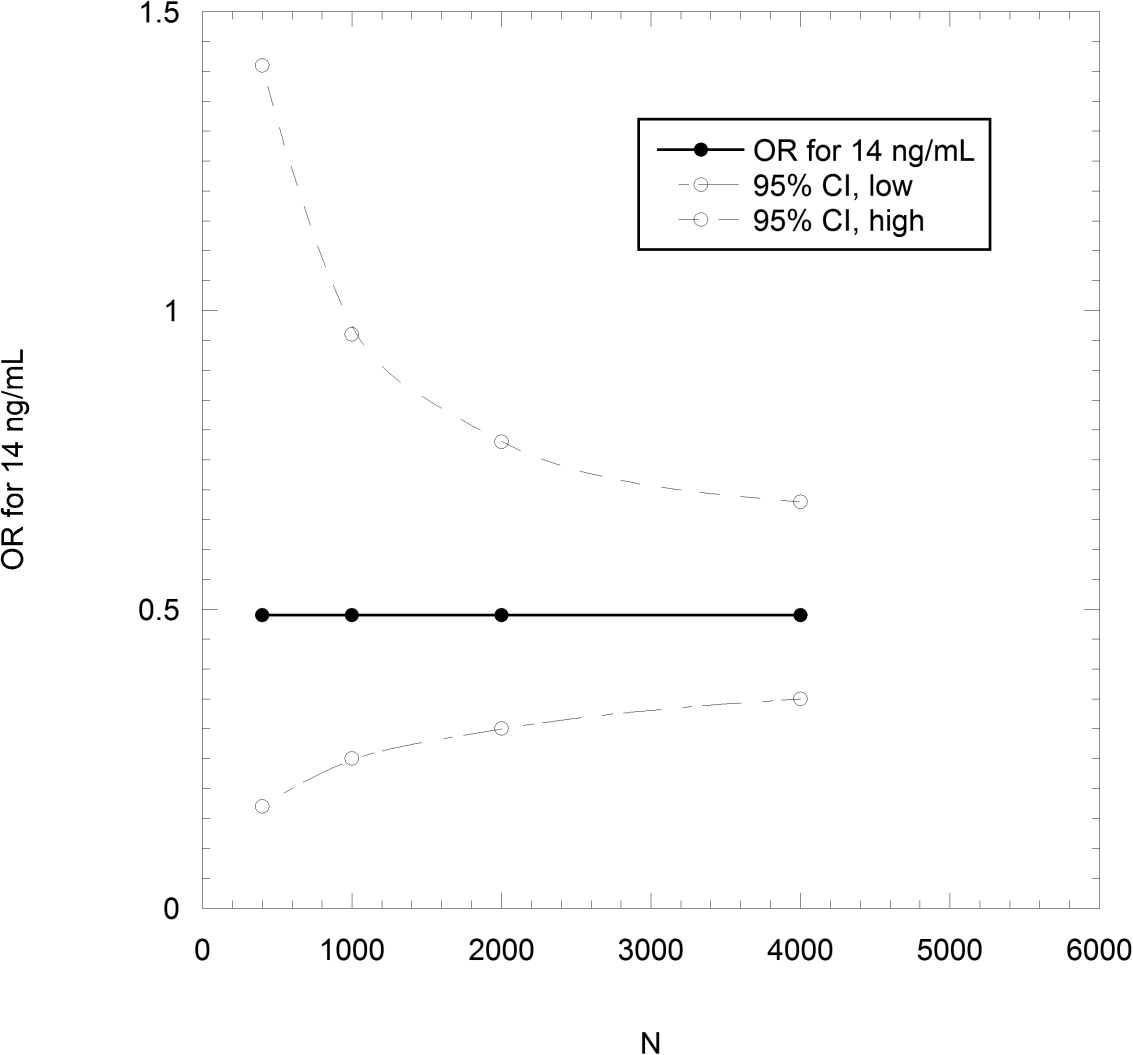
Odds ratio (OR) and 95% confidence interval (95% CI) as a function of the number of participants in the treatment and control arms, for vitamin D_3_ supplementation at 2000 IU/d for subjects with baseline 25-hydroxyvitamin D [25(OH)D] concentration of 14 ng/mL.

**Table 1:** Achieved 25(OH)D concentrations as a function of baseline 25(OH)D concentration and vitamin D supplementation for deciles of baseline 25(OH)D for Canadians aged 50–79 years, with increasing daily dose of vitamin D_3_.

Decile	25(OH)D 50¬79 years (ng/mL)	Achieved for 400 IU/d (ng/mL)	Achieved for 1000 IU/d (ng/mL)	Achieved for 2000 IU/d (ng/mL)	Achieved for 4000 IU/d (ng/mL)
1	11	25	29	31	45
2	14	18	24	33	47
3	17	21	28	35	49
4	20	24	29	37	50
5	23	27	32	40	51
6	26	29	35	42	53
7	29	33	37	44	55
8	33	36	40	47	57
9	36	39	43	49	59
10	40	43	46	52	61

25(OH)D = 25-hydroxyvitamin D.

All-cancer incidence rates reported for the U.S. population in 2005–2009 [23], and all-cancer incidence rate for those aged 65–69 years, 1760/100,000/yr, were used, and those values, together with the 25(OH)D concentration–breast cancer incidence relationship given above, and the achieved 25(OH)D concentrations for various daily vitamin D_3_ doses, [21] allow calculation of the number of cancer cases expected for various 25(OH)D concentrations (see Table 2).

**Table 2:** Number of cancer cases predicted by the current model for each baseline 25(OH)D concentration decile for those aged 50–79 years as a function of vitamin D supplementation dosage, assuming 4000 person-years (400 in each decile) in both treatment and control arms ratio (OR) = 18.3×[25(OH)D]^–0.833^.

Decile	25(OH)D 50¬79 years (ng/mL)	For baseline 25(OH)D_3_ (*N*)	For achieved 25(OH)D for 400 IU/d of vitamin D_3_ (*N*)	For achieved 25(OH)D for 1000 IU/d of vitamin D_3_ (*N*)	For achieved 25(OH)D for 2000 IU/d of vitamin D_3_ (*N*)	For achieved 25(OH)D for 4000 IU/d of vitamin D_3_ (*N*)
1	11	12.1	9.0	6.9	5.1	3.8
2	14	10.1	8.0	6.4	4.9	3.6
3	17	8.5	7.2	5.7	4.7	3.5
4	20	7.5	6.4	5.4	4.4	3.5
5	23	6.7	5.8	5.0	4.2	3.4
6	26	6.0	5.4	4.7	4.1	3.3
7	29	5.5	4.9	4.5	3.9	3.2
8	33	5.0	4.6	4.2	3.7	3.1
9	36	4.6	4.3	4.0	3.5	3.0
10	40	4.2	4.0	3.7	3.4	3.0
Sum		70.2	59.6	50.5	41.9	33.4

25(OH)D = 25-hydroxyvitamin D; *N* = number of cancer cases.

In this model, the equation for the number of cases is:

$$n_{i}=\;r\;x\;N_{i}\;x\;OR_{i}/\Sigma (OR_{i})$$

where n_i_ is the number of expected cancer cases for the n-th 25(OH)D concentration decile, r is the assumed population cancer incidence rate, N_i_ is the number of participant years in the n-th decile, OR_i_ is odds ratio calculated from the 25(OH)D concentration-breast cancer incidence rate relationship (as shown in Fig 1), and Σ_1-10_(OR_i_) is the sum of the OR_i_ for all ten deciles.

One can calculate the OR for cancer incidence, and their 95% CIs, using an online tool [http://www.vassarstats.net/odds2x2.html]. This tool uses as input variables the number of subjects found to develop the conditions of interest in the two arms of an RCT to calculate risk ratios and odds ratios along with their 95% confidence intervals. In our model, the condition is cancer incidence and the two groups in this case are the treatment and control arms.

## Results

**Fig 4:**
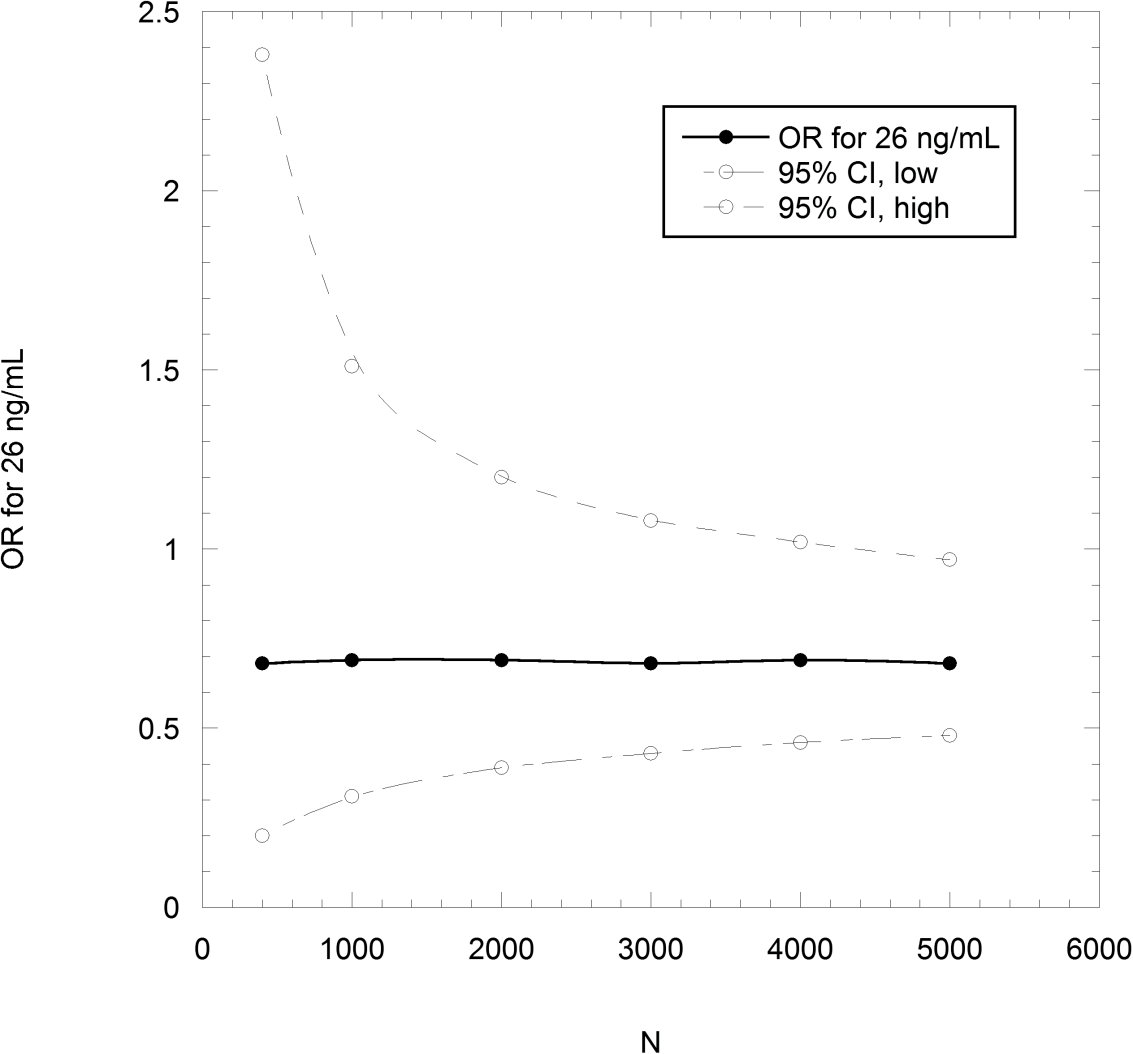
Odds ratio and 95% confidence interval (95% CI) as a function of the number of participants in the treatment and control arms for vitamin D_3_ supplementation at 2000 IU/d for subjects with baseline 25(OH)D concentration of 26 ng/mL.

The number, N, of participant years in the treatment and control arms to achieve a statistically significant reduction in all-cancer incidence is calculated from baseline and achieved 25(OH)D concentrations and expected cancer rates for these concentrations. The odds ratio is then calculated for a series of N values, and the results used to obtain a best-fit curve, in this case a smooth fit to the data—then, where the upper 95% CI value fit crosses the value 1.00 determines N. Thus, achieving a significant result for a baseline 25(OH)D concentration of 14 ng/mL and treatment with 2000 IU/d, resulting in an achieved 25(OH)D concentration of 34 ng/mL would take about 1000 participants each in the treatment and control arms (Fig 3). Starting with a baseline concentration of 26 ng/mL and achieving 42 ng/mL would increase the numbers per RCT arm to 4000 each (Fig 4).

The number of participant-years required [power needed] also varies depending on participant baseline vitamin D status [by upper or lower five deciles of 25(OH)D concentration in the Canadian population]. The approach described can be extended to calculate the number of participant years required for a range of baseline 25(OH)D concentrations by calculating the total number of cancer cases predicted for each pair of baseline and achieved concentration, for use in the odds ratio calculator. For example, ~1500 participant-years would be needed in each arm to achieve statistical significance for subjects in the lowest five deciles of baseline vitamin D status with 2000 IU/d vitamin D_3_ (Table 3), but ~10,000 participant-years in each arm for subjects with baseline status in the upper five deciles (Table 4). (Note that the calculations are based on the number of cancer cases per year; alternatively, the number of participants could be fixed and the number of cancer cases per year could be multiplied by the number of years.).

**Table 3:** Results of a model calculation using numbers of cases of breast cancer predicted by the proposed model for the lowest 5 deciles of vitamin D status, with increasing numbers of participant-years of supplementation at 2000 IU/d.

Decile	*N*	25(OH)D 50¬79 years (ng/mL)	Cases, control arm	Cases, treatment arm	RR (95% CI)
1	400	11	12.1	5.1	
2	400	14	10.1	4.9	
3	400	17	8.5	4.7	
4	400	20	7.5	4.4	
5	400	23	6.7	4.2	
Sum	1000		22.5	11.7	0.51 (0.25¬1.05)
	1500		33.7	17.5	0.51 (0.29¬0.92)
	2000		44.9	23.3	0.51 (0.31¬0.85)

25(OH)D = 25-hydroxyvitamin D; 95% CI = 95% confidence interval; *N* = number of participant-years in treatment and control arms; RR = relative risk.

**Table 4:** As in Table 3 but using five 25(OH)D deciles centered at 29 ng/mL and doses of 1000 IU/d of vitamin D_3_ for comparison with the results from a 2007 vitamin D RCT [13].

Decile	*N* placebo(266x3 yrs)	*N* vitDCa (403x3 yrs)	Baseline 25(OH)D 50¬79 years (ng/mL)	Cases, control arm	Cases, treatment arm	RR (95% CI)
5	160	242	23	2.7	2.8	
6	160	242	26	2.4	2.5	
7	160	242	29	2.2	2.3	
8	160	242	33	2.0	2.2	
9	160	242	36	1.8	2.1	
Total, 3 years	800	1210		11.1	11.9	0.70 (0.31¬1.60, P = 0.40)
Total, 4 years	1064	1610		14.8	15.9	0.71 (0.35¬1.43, P = 0.34)

25(OH)D = 25-hydroxyvitamin D; 95% CI = 95% confidence interval; *N* = number of participant-years in treatment and control arms; RR = relative risk, vitD, Ca, vitamin D plus calcium arms.

Two vitamin D RCTs report significant reductions in cancer incidence. The first was a 4-year trial involving postmenopausal women in Nebraska (Clinicaltrials.gov as NCT00352170) [13].The trial had three arms: 1100 IU/d of vitamin D_3_ plus 1450 mg/d of calcium, 1450 mg/d of calcium, and placebo, [mean baseline 25(OH)D concentration = 29 ng/mL]. The researchers analyzed results for all 4 years, and for the last 3 years. The justification for the second analysis was that some participants entering the study may have had undiagnosed cancer that was discovered during the first year. For the full 4-year period, the number of cancer cases was 20 in the placebo arm (n = 228), 17 in the calcium only arm (n = 445) and 13 in the vitamin D plus calcium arm (n = 446). The resulting relative risk for developing cancer on calcium alone was 0.53 (0.27–1.03) and 0.40 (0.20–0.80) in the vitamin D plus calcium arm. The ratio, 0.40/0.53 = 0.75 gives an estimate of the effect of vitamin D alone. For the final 3-year period, the cancer incidence was 18 in the placebo arm (n = 226), 15 in the calcium only arm (n = 416), and 8 in the vitamin D plus calcium arm (n = 403), with relative risks for developing cancer in the calcium only arm of 0.59 (0.29–1.24), and of 0.23 (0.09–0.60) in the vitamin D+calcium arm. The ratio, 0.23/0.59 = 0.39 provides an estimate of the effect of vitamin D alone. Our modeled estimate is a RR = 0.70 (0.31–1.60, P = 0.40) for the 3-years case and 0.71 (0.35–1.43, P = 0.34) for the 4-years case. This modeling result agrees more closely with their 4-year result than with their 3-year result even though our model result is not significant.

The second one was the Women’s Health Initiative trial (ClinicalTrials.gov number, NCT00000611), treatment arm used 400 IU/d of vitamin D_3_+1500 mg/d of calcium. No significant reduction in cancer incidence was apparent for the group overall. However, if analysis was restricted to participants who had not taken vitamin D or calcium supplements before entry, the hazard ratio was 0.86 (95% CI, 0.78–0.96), and significant [15]. Amongst those participants, 633 cancer cases occurred among the 7891 participant-years in the treatment arm and 715 cancer cases among the 7755 participant-years in the control arm. Comparing the model calculations with the results from that last trial is impossible because the distribution of 25(OH)D concentration among the participants is unknown. However, the finding is generally consistent with the model for the lower 25(OH)D deciles (Table 1).

In a vitamin D RCT of postmenopausal women in Nebraska (ClinicalTrials.gov Identifier:NCT01052051), participants took 2000 IU/d of vitamin D+1500 mg/d of calcium in the treatment arm or placebo. The mean baseline 25(OH)D concentration was 33±10 ng/mL, and rose to 44 ng/mL in the treatment arm. A total of 2064 participants completed the 4-year trial, with 45 cancer cases emerging in the treatment arm and 64 in the control arm, giving a hazard ratio of 0.70 (95% CI, 0.47–1.02, P = 0.06) [14]. Thus, treatment and control arms each encompassed ~8000 participant-years. Assuming those subjects came mostly from the upper five deciles of baseline 25(OH)D concentrations found in the Canadian data, the 8000 participant-years completed would, the present model suggests, result in a hazard ratio of 0.73 (0.48–1.12) (Table 5). Including calcium in the treatment, but not in the control arm, may have contributed to the suggestive result (discussed later).

**Table 5:** As in Table 3 but using the highest 5 deciles of vitamin D_3_ status and comparing results with those of a vitamin D RCT [14].

Decile	*N*	Baseline 25(OH)D 50¬79 years (ng/mL)	Cases, treatment arm	Cases, control arm	RR (95% CI)
6	400	26	4.1	6.0	
7	400	29	3.9	5.5	
8	400	33	3.7	5.0	
9	400	36	3.5	4.6	
10	400	40	3.4	4.2	
Sum	2000		18.6	25.3	0.73 (0.40¬1.34)
	4000		37.2	50.6	0.73 (0.48¬1.12)
	10,000		93.0	126.5	0.73 (0.56¬0.96)
*Memorandum*:					
Lappe reported (14)	4128	82	45	64	HR = 0.70, (0.47¬1.02, P = 0.06)

25(OH)D = 25-hydroxyvitamin D; 95% CI = 95% confidence interval; HR, hazard ratio; *N* = number of participant-years in treatment and control arms; RR = relative risk.

## Discussion

The results of this modeling study, inspired by Heaney’s guidelines [16], agree reasonably well with results from the Women’s Health Initiative trial and the recent Nebraskan trial [14], giving added confidence to both the model and those two RCT results.

Several observational studies and one RCT, suggest supplementation with vitamin D+calcium reduced cancer risk more than supplementation with vitamin D alone [24],[13],[25],[26]. Thus, to determine the effect of vitamin D alone, either the effect of calcium supplementation alone has to be determined if calcium supplementation is given solely to the treatment arm, as was the case in the later Lappe study [13], or both arms should receive calcium supplementation.

The findings using the present modeling system should put to rest two ideas. One is that RCTs have not shown vitamin D supplementation to reduce cancer risk, as several earlier reviews have suggested [11],[27]. The other is that vitamin D supplementation can yield no more than a 15% reduction in incidence of disease [28].

Ideally, vitamin D RCTs would start with a moderate loading dose, in deficiency, to ensure early rises in serum 25(OH)D concentrations, towards the planned target concentration [29]. The rationale for doing this, is that reaching the 25(OH)D plateau from supplementation with 1000 or 2000 IU/d of vitamin D_3_can take up to 4 months [30]. The ideal design would also allow for the fact that response to vitamin D supplementation is related not only to baseline 25(OH)D, which varies with season [20], but also to personal genetic factors [31],[32], body weight [33], and natural history of disease development, e.g. from early life [34]. In addition, investigators should consider including several 25(OH)D measurements for participants during the trial, [perhaps in late summer and late winter each year] to monitor the range of 25(OH)D achieved, together with compliance, and because, in long-term trials, 25(OH)D concentrations may vary with many other factors [35],[4]. Blood spot assays are inexpensive and convenient because they can be organized by mail [e.g. Heartland Assays LLC (Ames, IA) offers blood spot 25(OH)D assays using liquid chromatography–tandem mass spectrometry methodology- interassay coefficient of variation, 4.0% and intra-assay coefficient of variation<2.5%. [Andrew J. Makowski, Heartland Assays, LLC, personal communication, Jan. 30, 2017].

Many vitamin D_3_ RCTs aim to answer the question, ‘Does supplementing the healthy population with a modest amount of vitamin D affect health outcomes?’Because many of the large-scale trials underway rely on volunteers, they are likely to include people more interested in their health than the average person. Thus, those trials run the risk of being biased by the health of participants, as happened in observational studies on estrogen plus progestin use by postmenopausal women for cardiovascular disease risk reduction which supported their use, but a large-scale RCT found adverse effects, such as increased risk of cancer, that countered the benefits [36].

Although the present modeling study was based on cancer outcomes, and the modeling findings agree reasonably well with the two positive vitamin D RCTs for cancer, it could also provide the basis for designing vitamin D RCTs for other health outcomes [see Appendix]. Many health outcomes have similar target 25(OH)D concentrations [37], and we suggest that the goal of vitamin D RCTs should be to elucidate the relationship between baseline and achieved 25(OH)D concentrations, and health outcomes, using data from observational studies as a basis for RCT planning [38],[4].

While the modeling suggested should contribute to designing, and analyzing, vitamin D RCT data for many health outcomes, this approach depends on reliable, accurate, reproducible and comparable 25(OH)D assays, with harmonization of findings where different assay systems are used, all of which are now achievable [39],[40],[41].

Several large-scale vitamin D RCTs underway will examine how vitamin D supplementation affects cancer incidence or progression (see, e.g., Table 3 in [42]). They may or may not have measured 25(OH)D concentration at enrollment, but if they did, they did not seek to enroll participants with low 25(OH)D concentrations. However, the approach used in this modeling study could prove useful in analyzing and interpreting the data those RCTs produce.

One might argue that basing analyses on circulating 25(OH)D concentrations, rather than vitamin D dosing, changes RCTs into observational studies, but the use of the suggested modeling methodology allows the use of 25(OH)D concentrations to improve targeting of supplementation to baseline status, and to ensure achievement of target vitamin D status. Although this additional definition of RCT conditions would complicate the conduct and analysis of RCTs, it would in no way change the fact that RCTs organized with this additional methodology would be interventional, while adding considerably to the ability of vitamin D RCTs to be able to detect causality.

## Appendix

Examples of conditions where data exist for use in management and analyses of RCTs of vitamin D for using the proposed modeling for chronic, non-cancerous conditions, and where ‘classic’ RCT analysis showed no benefits, include (1), insulin resistance, where earlier RCT data showed IR was reduced, but only reached significance with achieved serum 25(OH)D values ≥32 ng/mL after at least 6 months in deficient south Asians [43]; (2), preterm birth rate reductions were shown in South Carolina when data was examined by maternal vitamin D status within 6 weeks of birth, plateauing at ~40 ng/ml, though no benefits were found on analysis by vitamin D intakes [44],[45]; (3), no reductions in pre-eclampsia were found in a RCT in 3 American centers giving 4400 IU/day vs. 400 IU/day of vitamin D_3_ (for reduction of offspring atopic disease) when analyzed by intention to treat. Low maternal vitamin D status was, however, a strong predictor of later pre-eclampsia, and pre-eclampsia risk was significantly reduced once achieved maternal serum 25(OH)D reached ≥70 ng/ml [46].
